# Sulodexide Decreases Albuminuria and Regulates Matrix Protein Accumulation in C57BL/6 Mice with Streptozotocin-Induced Type I Diabetic Nephropathy

**DOI:** 10.1371/journal.pone.0054501

**Published:** 2013-01-22

**Authors:** Susan Yung, Mel K. M. Chau, Qing Zhang, Chen Zhu Zhang, Tak Mao Chan

**Affiliations:** Department of Medicine, University of Hong Kong, Queen Mary Hospital, Pokfulam, Hong Kong; Children's Hospital Boston, United States of America

## Abstract

**Objective:**

Sulodexide is a mixture of glycosaminoglycans that may reduce proteinuria in diabetic nephropathy (DN), but its mechanism of action and effect on renal histology is not known. We investigated the effect of sulodexide on disease manifestations in a murine model of type I DN.

**Methods:**

Male C57BL/6 mice were rendered diabetic with streptozotocin. After the onset of proteinuria, mice were randomized to receive sulodexide (1 mg/kg/day) or saline for up to 12 weeks and renal function, histology and fibrosis were examined. The effect of sulodexide on fibrogenesis in murine mesangial cells (MMC) was also investigated.

**Results:**

Mice with DN showed progressive albuminuria and renal deterioration over time, accompanied by mesangial expansion, PKC and ERK activation, increased renal expression of TGF-β1, fibronectin and collagen type I, III and IV, but decreased glomerular perlecan expression. Sulodexide treatment significantly reduced albuminuria, improved renal function, increased glomerular perlecan expression and reduced collagen type I and IV expression and ERK activation. Intra-glomerular PKC-α activation was not affected by sulodexide treatment whereas glomerular expression of fibronectin and collagen type III was increased. MMC stimulated with 30 mM D-glucose showed increased PKC and ERK mediated fibronectin and collagen type III synthesis. Sulodexide alone significantly increased fibronectin and collagen type III synthesis in a dose-dependent manner in MMC and this increase was further enhanced in the presence of 30 mM D-glucose. Sulodexide showed a dose-dependent inhibition of 30 mM D-glucose-induced PKC-βII and ERK phosphorylation, but had no effect on PKC-α or PKC-βI phosphorylation.

**Conclusions:**

Our data demonstrated that while sulodexide treatment reduced proteinuria and improved renal function, it had differential effects on signaling pathways and matrix protein synthesis in the kidney of C57BL/6 mice with DN.

## Introduction

Diabetic nephropathy (DN) is a leading cause of end-stage renal disease in developed countries. DN is characterized by glomerular hypertrophy, basement membrane thickening, disruption of the glomerular permeability barrier, progressive accumulation of glomerular matrix, culminating in glomerulosclerosis, tubulo-interstitial fibrosis, and progressive proteinuria and deterioration of renal function [1]–[4].

The extracellular matrix (ECM) plays an active role in regulating the structure and function of adjacent cells, and influences cell morphology, differentiation, anchorage and intercellular communication [5], [6]. Alterations in the ECM is a prominent feature in DN. Data from animal and *in vitro* studies have demonstrated that TGF-β1 can up-regulate matrix protein synthesis, and it plays a pivotal role in the hypertrophic and fibrotic manifestations of DN [7]–[9]. Perlecan is a heparan sulfate proteoglycan that maintains normal glomerular basement membrane (GBM) structure [10], [11], and is involved in the transport of cells and small molecules across the GBM. Perlecan also binds cytokines and growth factors through its glycosaminoglycan chains, thereby acting as a reservoir for these peptides and preventing them from being degraded. Perlecan is a major contributor to the perm-selectivity of the GBM and a reduction of these negatively charged macromolecules results in proteinuria [12].

Mesangial cells constitute up to 40% of the total cells in the glomerulus. They occupy a central position in the kidney where they play a critical role in renal homeostasis and physiology, and provide structural support to the glomerular capillary loops [13]. Mesangial cells are embedded in their own matrix, which they synthesize and remodel. Qualitative and quantitative changes in the mesangial matrix will have a profound effect on mesangial cell function.

Current treatment for DN, such as glycaemic and blood pressure control and intervention of the renin-angiotensin pathway using ACE inhibitors or angiotensin II receptor antagonists [14], [15], are at best partially effective. The majority of patients progress on a relentless course of renal failure. The quest for new treatments thus remains an unmet need. Sulodexide is a mixture of glycosaminoglycans with 80% fast-moving heparin and 20% dermatan sulfate [16], [17]. It bears strong chemical similarities to heparin but does not have anti-coagulation properties when given orally. Treatment with sulodexide has been shown to reduce proteinuria in patients with DN [18]–[20]. However, data from a recent controlled trial showed negative results [21]. In the present study, we investigated the effect of sulodexide on renal histopathology and disease phenotype in a murine model of type I DN, and its effects on fibrogenic processes in mesangial cells.

We demonstrated that sulodexide improved proteinuria and renal function in mice with DN, which was associated with increased perlecan expression along the GBM. Furthermore, sulodexide selectively decreased renal expression of collagen type I and IV, but increased glomerular expression of fibronectin and collagen type III.

## Materials and Methods

### Ethics Statement

All animal procedures were approved by the Institutional Committee on the Use of Live Animals in Teaching and Research at the University of Hong Kong.

### Chemicals and Reagents

All chemicals and reagents were of the highest purity and purchased from Sigma-Aldrich Chemical Company (Tin Hang Technology Ltd, Hong Kong) unless otherwise stated. Antibodies to perlecan, collagen type I, collagen type III, collagen type IV, TGF-β1 and phosphorylated PKC-α and PKC-βI were purchased from Santa Cruz Biotechnology Inc. (Genetimes Technology International Holding Ltd, Hong Kong). Phosphorylated PKC-βII and ERK were purchased from Cell Signaling Technology (Gene Company, Hong Kong). Fibronectin antibody was purchased from Sigma-Aldrich Chemical Company (Tin Hang Technology Ltd, Hong Kong). QuantiChrom albumin, creatinine and urea assay kits were purchased from BioAssay Systems (California, USA). Accu-Chek Advantage II Glucostix test strips and Accu-Chek Advantage blood glucose meter were purchased from Roche Diagnostics (DKSH Hong Kong Ltd, Hong Kong). Sulodexide (Vessel Due F) was purchased from Alfa Wassermann (Guangzhou, China).

### Animal Studies

Male C57BL/6 mice at 6–8 weeks of age were purchased from the Laboratory Animal Unit (University of Hong Kong, Hong Kong) and received standard chow and water *ad libitum.* After one week acclimatizing to their surroundings, mice were fasted for 6 h prior to intra-peritoneal injection of streptozotocin (STZ, 50 mg/kg) in 10 mM citrate buffer, pH 4.5, administered on five consecutive days. Diabetes mellitus was confirmed by tail vein blood sampling of glucose concentration, measured with Accu-Chek Advantage II Glucostix test strips. Spot urine was tested weekly for albuminuria with QuantiChrom albumin assay kit until sacrifice. Mice with elevated blood glucose levels (>10 mM) and albuminuria (>100 mg/dl) on two separate occasions two days apart (defined as ‘baseline’ in the animal studies) were randomized to receive treatment with either saline (vehicle control) or sulodexide (1 mg/kg/day) by oral gavage for 2, 4, 8 or 12 weeks (6 mice per time-point for each group). After 2, 4, 8 and 12 weeks of treatment, mice were sacrificed, blood samples were obtained by cardiac puncture and the kidneys harvested, decapsulated and weighed. The left kidney was cut perpendicular to the long-axis and one half of the kidney was snap frozen in OCT followed by immersion in liquid nitrogen, while the second half was fixed in 10% neutral-buffered formalin followed by paraffin embedding. Renal cortical tissue from the right kidney was separated from the medulla and frozen at −80°C until mRNA isolation. Six diabetic mice that had just developed proteinuria were also sacrificed to obtain baseline values for clinical, histological and morphometrical parameters. Negative control groups included non-diabetic male C57BL/6 mice treated with either saline or sulodexide for 12 weeks. Serum creatinine and urea levels were measured using QuantiChrom creatinine and urea assay kits respectively.

### Histological Assessment of the Kidney

Paraffin-embedded kidney sections (5 µm) were stained with periodic acid-Schiff (PAS) and Masson’s trichrome for histologic and morphometric analysis. Thirty cross-sectional profiles of PAS-stained glomeruli were captured for each mouse. The glomerular tuft area was determined using Axiovision 4.3 software (Zeiss, Hong Kong). Assessment of mesangial matrix accumulation, denoted by PAS-positive staining in nuclei-free areas of the mesangium, was assessed in 30 randomly selected glomeruli and scored in a blinded manner on a scale of 0 to 4, where 0 = 0–5%, 1 =  >5–25%, 2 =  >25–50%, 3 =  >50–75%, 4 =  >75% deposition. The scores reflected variations in the extent rather than intensity of staining, and the reproducibility of this scoring system has been documented [22]. The ‘sclerotic index’ referred to the mean score. Collagen deposition was assessed with Masson’s trichrome staining of 30 glomeruli, scored in a blinded manner using the aforementioned system and expressed as an arbitrary unit (AU). Tubulo-interstitial changes such as collagen deposition, tubular dilation and/or atrophy, and inflammatory cell infiltration were assessed in 10 non-overlapping areas free of glomeruli, and graded on a scale 1–4, where 0 =  normal, 0.5 =  small focal areas of damage, 1 =  <10%, 2 = 10–25%, 3 = 26–75% and 4 =  >75% damage in the renal cortex [22] and expressed as mean tubulo-interstitial score for each group.

### Cytochemical Staining

Detection of phosphorylated ERK and PKC-α, TGF-β1, fibronectin, and collagen type I, III and IV in paraffin-embedded kidney sections (5 µm) from non-diabetic or DN mice treated with saline or sulodexide was performed with specific antibodies (dilution 1∶50), followed by peroxidase-anti-peroxidase staining, counterstained with hematoxylin and examined with an Axioskop 2 plus microscope as previously described [23], [24]. Staining in the capillary loops, mesangium and tubulo-interstitium was semi-quantitatively assessed in at least 30 glomeruli and tubules per mouse kidney, and the extent of staining graded as follows: 0 = 0–5% staining, 1 =  >5–25% staining, 2 =  >25–50% staining, 3 =  >50–75% staining, 4 =  >75% staining [22].

### Immunohistochemical Analysis of Perlecan Expression

Snap frozen renal sections (8 µm) were blocked with 3% BSA in PBS, incubated with rabbit anti-mouse perlecan antibody followed by the appropriate secondary antibody in a darkened humidified chamber. All incubation periods were 1 h at 37°C and sections were washed thrice with PBS between steps. Sections were mounted with fluorescent mountant and epifluorescence viewed using an Axioskop 2 plus fluorescent microscope. Images of 30 randomly selected glomeruli from control and treated mice were captured and scored in a blinded manner as described above.

### Quantitative Real-time Polymerase Chain Reaction for TGF-β1, Fibronectin, Collagen Type I, III and IV, Perlecan and Heparanase mRNA

RNA was isolated from snap frozen renal cortical tissue from control and treated mice using Ambion® ToTALLY RNA™ Total RNA isolation kit according to the manufacturer’s instructions. One microgram of total RNA was reverse transcribed to cDNA with M-MLV transcriptase using the random hexamers method. Taqman quantitative real-time PCR reactions was performed in duplicate using primer sets for TGF-β1, fibronectin, collagen type I, collagen type III, collagen type IV, perlecan and heparanase according to the manufacturer’s instructions (Assays-on-Demand ID: Mm00441726_m1 for TGF-β1, Mm00692666_m1 for fibronectin, Mm00801666_g1 for collagen type I, Mm01254478_g1 for collagen type III, Mm01210125_m1 for collagen type IV, Mm01181165_m1 for perlecan and Mm00461768_m1 for heparanase, Applied Biosystems, Hong Kong) in a Lightcycler 480 II real-time PCR system. Comparative real-time PCR results normalized to GAPDH were analyzed using the Lightcycler 480 Software vs 1.5.0SP3 (Roche Diagnostics, DKSH Hong Kong Limited, Hong Kong).

### Culture of Murine Mesangial Cells (MMC)

MMC from BALB/c mice were obtained by differential sieving of glomeruli and collagenase digestion. Cells were cultured in RPMI 1640 medium containing 10% FCS and characterized by their stellate morphology, ability to form hillocks, and immunohistochemical staining (positive for vimentin and negative for cytokeratin and von Willebrand Factor). All experiments were conducted on MMC of the 7–10^th^ passage that had been growth arrested for 72 h. MMC were pre-conditioned with 5 mM D-glucose (physiological concentrations), 30 mM D-glucose or 30 mM mannitol (osmotic control) for 1 week before experiments. Following pre-conditioning with glucose or mannitol, MMC were cultured with 5 mM or 30 mM D-glucose, or 30 mM mannitol, in the presence or absence of sulodexide (0–200 µg/ml) for 24 h, and matrix protein synthesis and phosphorylation of ERK, PKC-α, PKC-βI and PKC-βII were then investigated. Specific inhibitors to PKC (Gö6976, 10 µM) and ERK (PD98059, 50 µM) were used to determine whether fibronectin and collagen type III synthesis was mediated through PKC or ERK phosphorylation.

### Western Blot Analysis

Whole cell lysates of MMC were obtained by solubilizing cells cultured under control or experimental conditions in 20 mM sodium acetate (pH 6.0) containing 4 M urea and 1% Triton X-100 (200 µl). Aliquots of each cell lysate (10 µg total protein content determined with a modified Lowry assay) were denatured in sample buffer at 95°C for 5 min and subjected to SDS-PAGE. Samples were electrophoresed on 8% acrylamide gels to investigate fibronectin and collagen type I and III synthesis, and on 12% acrylamide gels to investigate ERK, PKC-α, PKC-βI and PKC-βII phosphorylation [24]. Proteins were transferred onto nitrocellulose membranes using a mini-gel transfer system at 100 V for 1 h at 4°C. Equal loading of proteins was confirmed by staining the membranes with Ponceau S solution. Membranes were immunoblotted with primary antibodies to fibronectin, collagen type III, β-actin, total and phosphorylated (phospho) ERK, PKC-α, PKC-βI and PKC-βII, followed by the relevant horseradish peroxidase-conjugated secondary antibodies as previously described [25]. Bands were visualized by ECL and the band intensity semi-quantitated by densitometry using ImageJ (NIH) software, normalized to their respective house-keeping protein and expressed as arbitrary densitometric unit (DU). Fibronectin and collagen type III were normalized to β-actin, and phospho-ERK, phospho-PKC-α, phospho-PKC-βI and phospho-PKC-βII were normalized to total ERK, PKC-α, PKC-βI and PKC-βII respectively.

### Statistical Analyses

Results are expressed as mean+SD. Statistical analysis was performed using GraphPad Prism version 5.0 for Windows, (GraphPad Software, San Diego, CA, USA). Differences were assessed by ANOVA followed by Bonferroni’s multiple comparison post-test. Two-tailed *P*<0.05 was considered statistically significant.

## Results

Persistent proteinuria accompanied by blood glucose level above 30 mM was observed in 50% of male C57BL/6 mice 4–5 weeks after intra-peritoneal STZ administration. These mice were then randomized to either saline or sulodexide treatment for periods up to 12 weeks, with non-diabetic mice under same treatments as negative controls.

### Sulodexide Reduces Albuminuria and Renal Function Deterioration in DN Mice

**Figure 1:**
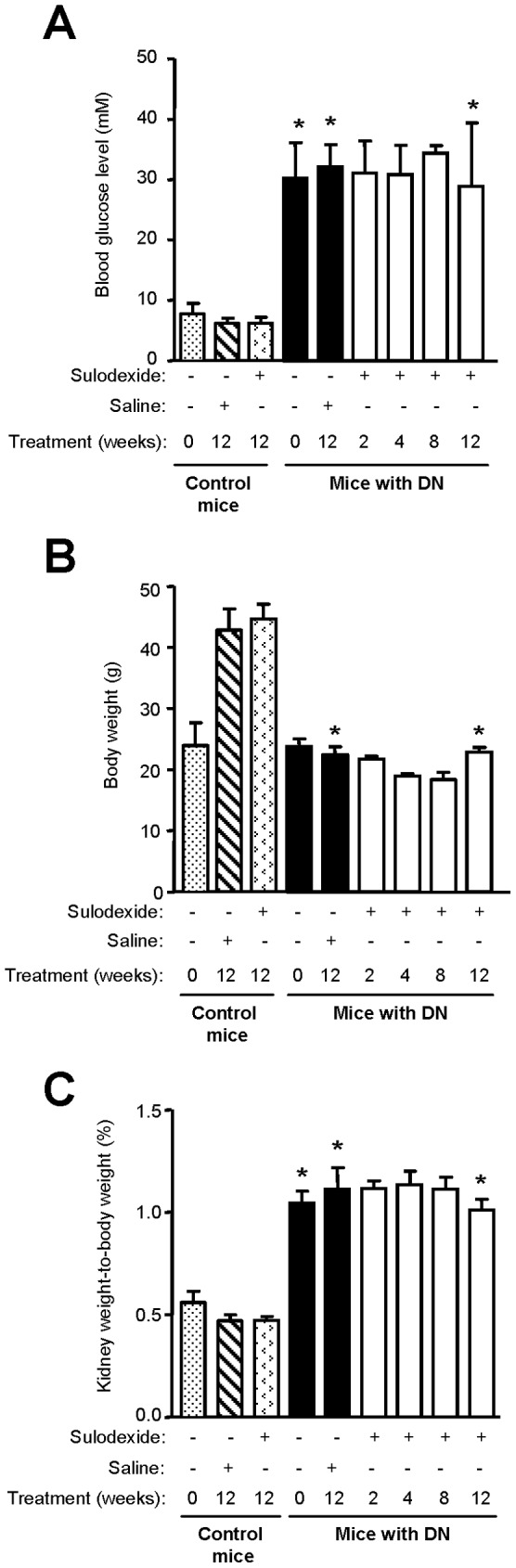
The effect of sulodexide on blood glucose, body weight, and kidney weight-to-body weight ratio in control and DN C57BL/6 mice. (A) Blood glucose level, (B) body weight and (C) kidney weight-to-body weight ratio in control and DN mice treated with saline or sulodexide are shown. Results are expressed as mean+SD of data obtained from 6 mice per group. DN, diabetic nephropathy. *P<0.001, with vs without DN for the same time-point.

There was no difference in the survival of saline- or sulodexide-treated DN or non-diabetic mice (data not shown). Elevated blood glucose level remained stable over time in sulodexide-treated DN mice, and was comparable to saline-treated controls (Figure 1A). Sulodexide did not affect the blood glucose level in non-diabetic mice. Mice with DN failed to gain weight, and their weight was 49.80% that of their non-diabetic counterpart after 12 weeks (22.43±3.32 and 44.68±5.91 g respectively, *P*<0.001) (Figure 1B). The kidney weight-to-body weight ratio was significantly higher in DN mice compared with non-diabetic mice (1.11±0.26% vs 0.47±0.04% after 12 weeks, *P*<0.001) (Figure 1C). Sulodexide treatment did not affect body weight or kidney weight-to-body weight ratio (Figure 1B and C).

**Figure 2:**
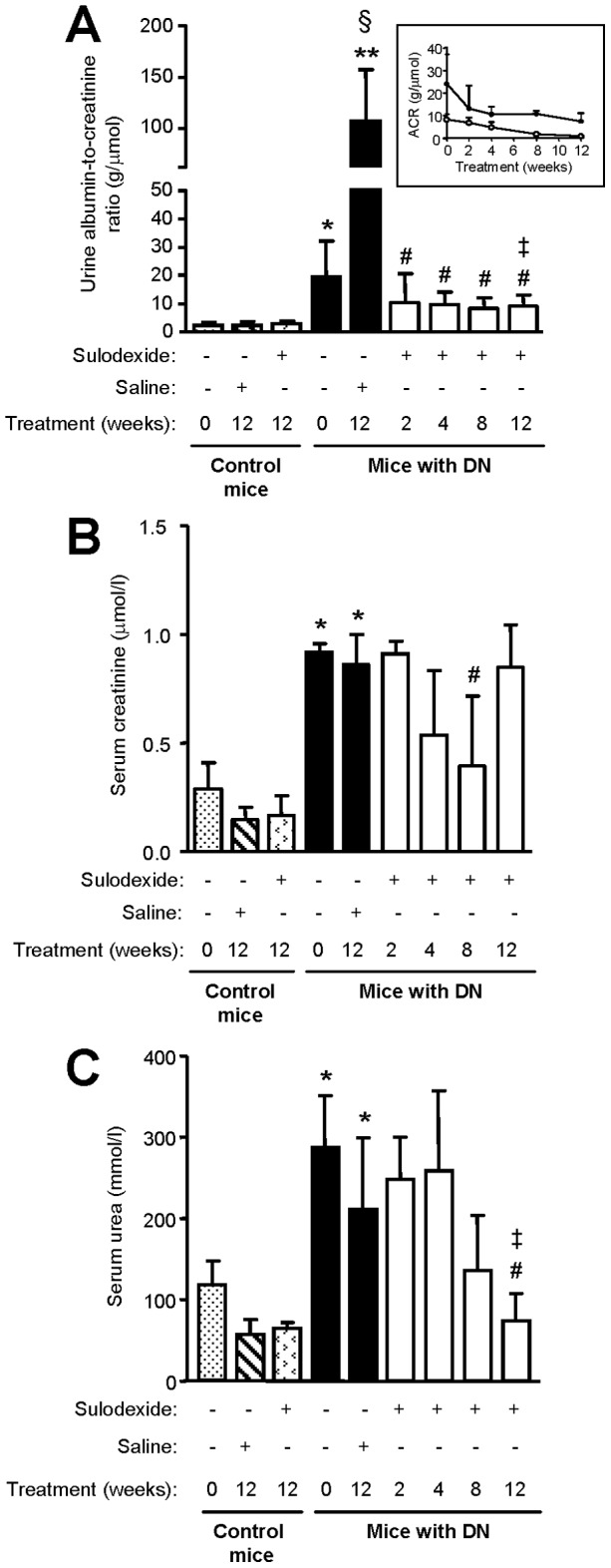
The effect of sulodexide on albuminuria and renal function in control and DN C57BL/6 mice. (A) Urine albumin-to-creatinine (ACR) ratio, (B) serum creatinine level and (C) serum urea level in control and DN mice treated with saline or sulodexide are shown. Results are expressed as mean+SD of data obtained from 6 mice per group. Insert in (A) shows the effect of sulodexide on ACR in mice with microalbuminuria (white circle) and macroalbuminuria (black circle) with time. *P<0.01, **P<0.001, with vs without DN for the same time-point, ^#^ *P*<0.01, DN baseline vs sulodexide-treated DN mice, ^§^ *P*<0.001, DN baseline vs saline-treated DN mice, ^‡^ *P*<0.01, saline vs sulodexide treatment for the same time-point.

Urine albumin-to-creatinine ratio (ACR) increased over time in DN mice (105.30±51.47 vs 19.42±12.65 g/µmol, 12 weeks vs baseline, *P*<0.001), which was markedly reduced with sulodexide treatment (Figure 2A). After 12 weeks of sulodexide treatment, urine ACR was reduced by 53.86% compared with baseline (*P*<0.01) and 91.40% compared with saline-treated DN mice (*P*<0.001). The proteinuria reducing effect of sulodexide was observed when baseline albuminuria was 100 mg/dl or above 300 mg/dl (Figure 2A). Serum creatinine and urea levels were significantly higher in DN mice when albuminuria became manifest compared with non-diabetic mice of the same age (*P*<0.001). Both were significantly reduced after sulodexide treatment (Figure 2B and C).

### Effect of Sulodexide on Renal Histology

**Figure 3:**
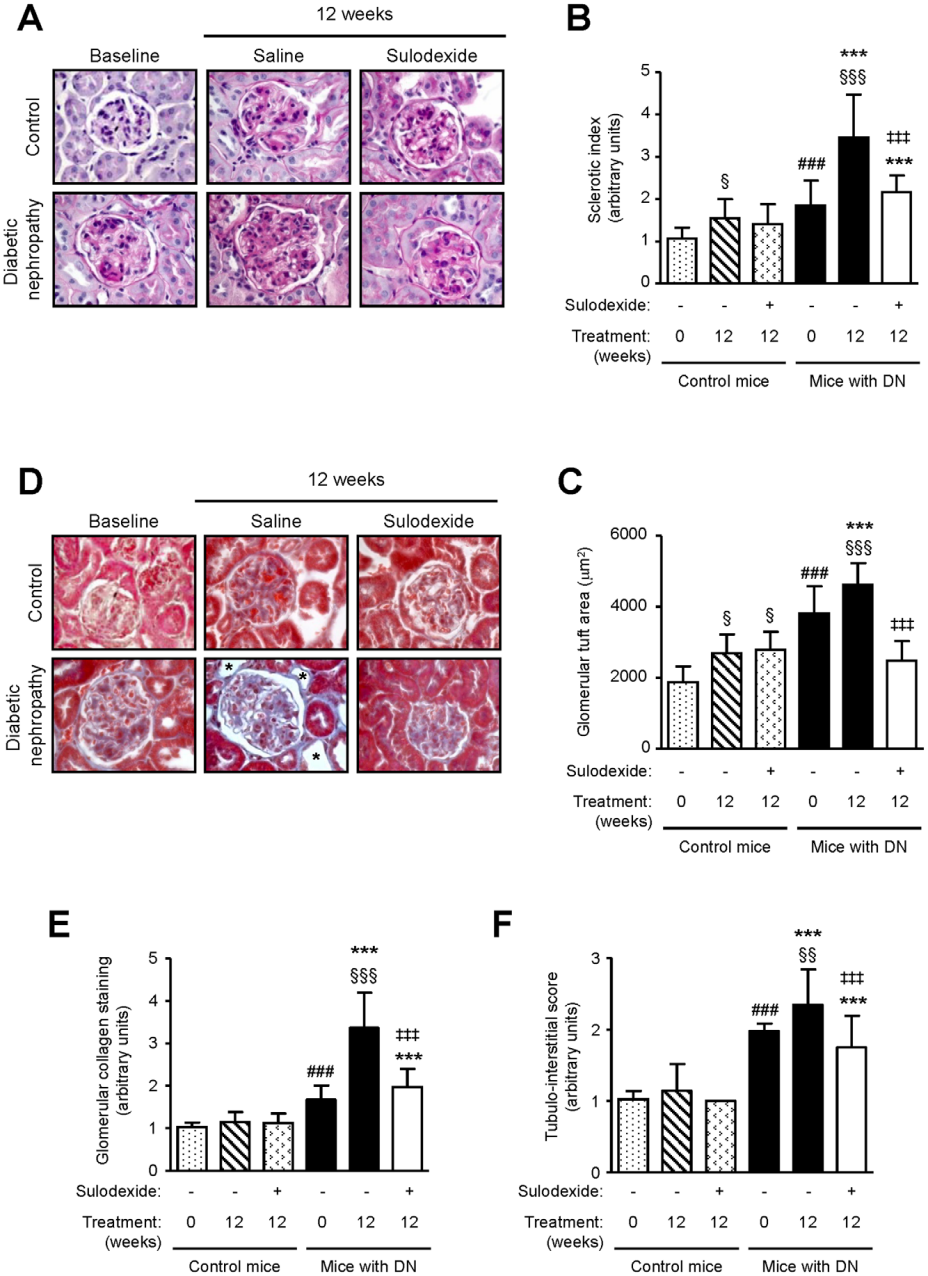
The effect of sulodexide treatment on renal histology in control and DN C57BL/6 mice. (A) Representative images of PAS stained renal specimens obtained from control and DN mice at baseline and after 12 weeks treatment with saline or sulodexide are shown. Mesangial expansion, thickening of the Bowman’s capsule, and increased matrix accumulation are observed in DN mice and these changes are reduced in mice treated with sulodexide, to levels similar to those observed in non-diabetic mice. Image-based computer assisted analysis was performed to semi-quantify (B) glomerular sclerotic index as determined by the extent of PAS-positive staining and (C) surface area of the glomerular tuft. (D) Representative images of renal sections stained for collagen deposition using Masson’s trichrome staining (depicted by the blue color). Asterisks denote tubular atrophy. Image-based computer assisted analysis was performed to semi-quantify (E) the amount of collagen deposition in the glomeruli and (F) tubulo-interstitial changes in control and DN mice. Results are expressed as mean+SD of data obtained from 6 mice per group. ***P<0.001, DN mice vs non-diabetic mice for the same treatment at the same time-point, ^###^ *P*<0.001, DN baseline vs non-diabetic baseline, ^§^ *P*<0.05, ^§§^ *P*<0.01, ^§§§^ *P*<0.001, compared to baseline for the same group, ^‡‡‡^ *P*<0.001, saline vs sulodexide for the same time-point in DN mice. Original magnification x1000 for panels (A) and (D).

Glomerular abnormalities in DN mice were evident at the onset of proteinuria, and became more severe over time. These included increased glomerular surface area, mesangial expansion, thickening of the GBM and Bowman’s capsule, and increased deposition of matrix proteins within the mesangial matrix (Figure 3A–E). The ‘sclerotic index’, which reflects glomerular matrix accumulation, increased over time in saline-treated DN mice (3.45±1.02 vs 1.85±0.59, 12 weeks vs onset of albuminuria, *P*<0.001) (Figure 3A and B), but was reduced with sulodexide treatment (2.15±0.40 after 12 weeks) (*P*<0.001) (Figure 3B). The reduction in ‘sclerotic index’ was accompanied by a significant reduction in glomerular area (Figure 3A and C). Collagen deposition in the glomerulus was markedly reduced following sulodexide treatment (Figure 3D and E). Tubulo-interstitial changes such as tubular atrophy and deposition of collagen in the interstitium were noted in saline-treated mice after 12 weeks and were markedly reduced in sulodexide-treated mice (Figure 3D and F).

### Effect of Sulodexide on Perlecan and Heparanase Expression in DN Mice

**Figure 4:**
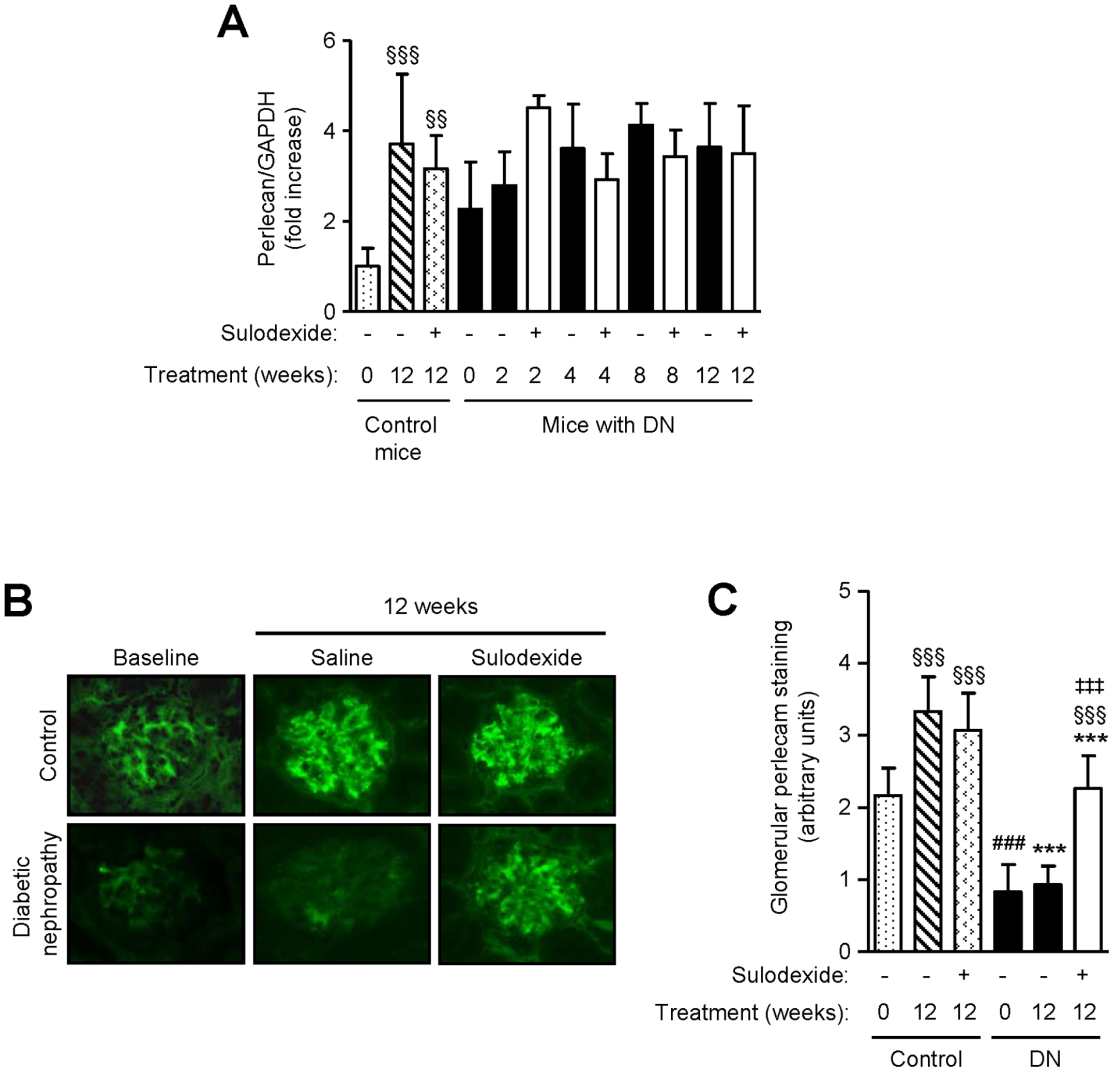
The effect of sulodexide on perlecan expression in renal tissue in control and DN C57BL/6 mice. (A) Gene expression of perlecan core protein in control and DN mice treated with saline or sulodexide as determined by real-time PCR. (B) Representative images of perlecan expression in snap frozen renal tissue from control and DN mice at baseline and after 12 weeks treatment with saline or sulodexide are shown. Original magnification x1000. (C) Image-based computer assisted analysis was performed to semi-quantify the amount of perlecan in the glomeruli of control and DN mice. Results are expressed as mean+SD of data obtained from 6 mice per group. ^§§§^ *P*<0.001, compared to baseline for the same group, ^###^ *P*<0.001, DN baseline vs non-diabetic baseline, ***P<0.001, DN mice vs non-diabetic mice for the same treatment, ^‡‡‡^ *P*<0.001, saline vs sulodexide treatment for the same time-point in DN mice.

Perlecan is a heparan sulfate proteoglycan that plays a critical role in the perm-selectivity of the GBM. Sulodexide treatment did not affect perlecan mRNA or core protein expression in non-diabetic mice (Figure 4A–C). Glomerular perlecan core protein was markedly reduced in DN mice compared with non-diabetic controls, and was partly restored after sulodexide treatment (Figure 4A–C). Weak staining of perlecan was noted in the tubulo-interstitial compartment of the kidney in control and sulodexide-treated mice (Figure 4B).

**Figure 5:**
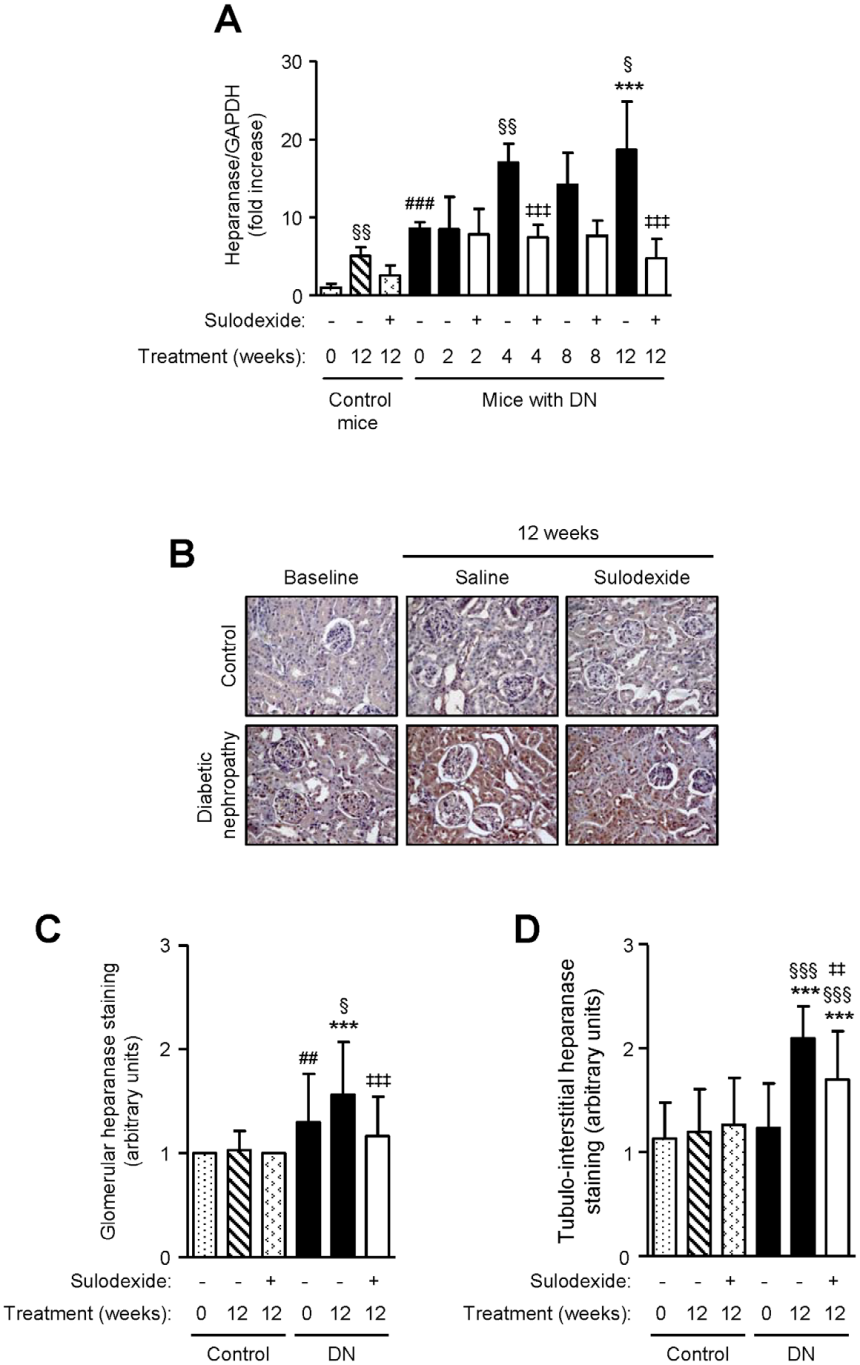
The effect of sulodexide on heparanase expression in renal tissue in control and DN C57BL/6 mice. (A) Gene expression of heparanase in control and DN mice treated with saline or sulodexide as determined by real-time PCR. (B) Representative images of heparanase protein expression in control and DN mice at baseline and after 12 weeks treatment with saline or sulodexide are shown. Original magnification x1000. Image-based computer assisted analysis was performed to semi-quantify the amount of heparanase in the (C) glomeruli and (D) tubulo-interstitium of control and DN mice. Results are expressed as mean+SD of data obtained from 6 mice per group. ^§^ *P*<0.05, ^§§^ *P*<0.01, ^§§§^ *P*<0.001, compared to baseline for the same group, ^##^ *P*<0.01, ^###^ *P*<0.001, DN baseline vs non-diabetic baseline, ***P<0.001, DN mice vs non-diabetic mice for the same treatment, ^‡‡^ *P*<0.01, ^‡‡‡^ *P*<0.001, saline vs sulodexide treatment for the same time-point in DN mice.

Heparanase is increased in patients with DN, which degrade heparan sulfate glycosaminoglycan chains thereby reducing the electronegativity of the GBM and contributing to proteinuria [26]. DN mice showed a progressive increase in heparanase mRNA level, which was 3.89-folds higher than that of non-diabetic controls after 12 weeks (Figure 5A), and was accompanied by a concomitant increase in heparanase protein expression in the glomeruli and tubulo-interstitium (Figure 5B–D). Sulodexide treatment significantly decreased heparanase mRNA in DN mice to levels similarly observed in non-diabetic mice after 12 weeks (Figure 5A), and this was associated with a decrease in heparanase protein expression in both compartments of the kidney (Figure 5B–D).

### Effect of Sulodexide on PKC-α and ERK Phosphorylation

**Figure 6:**
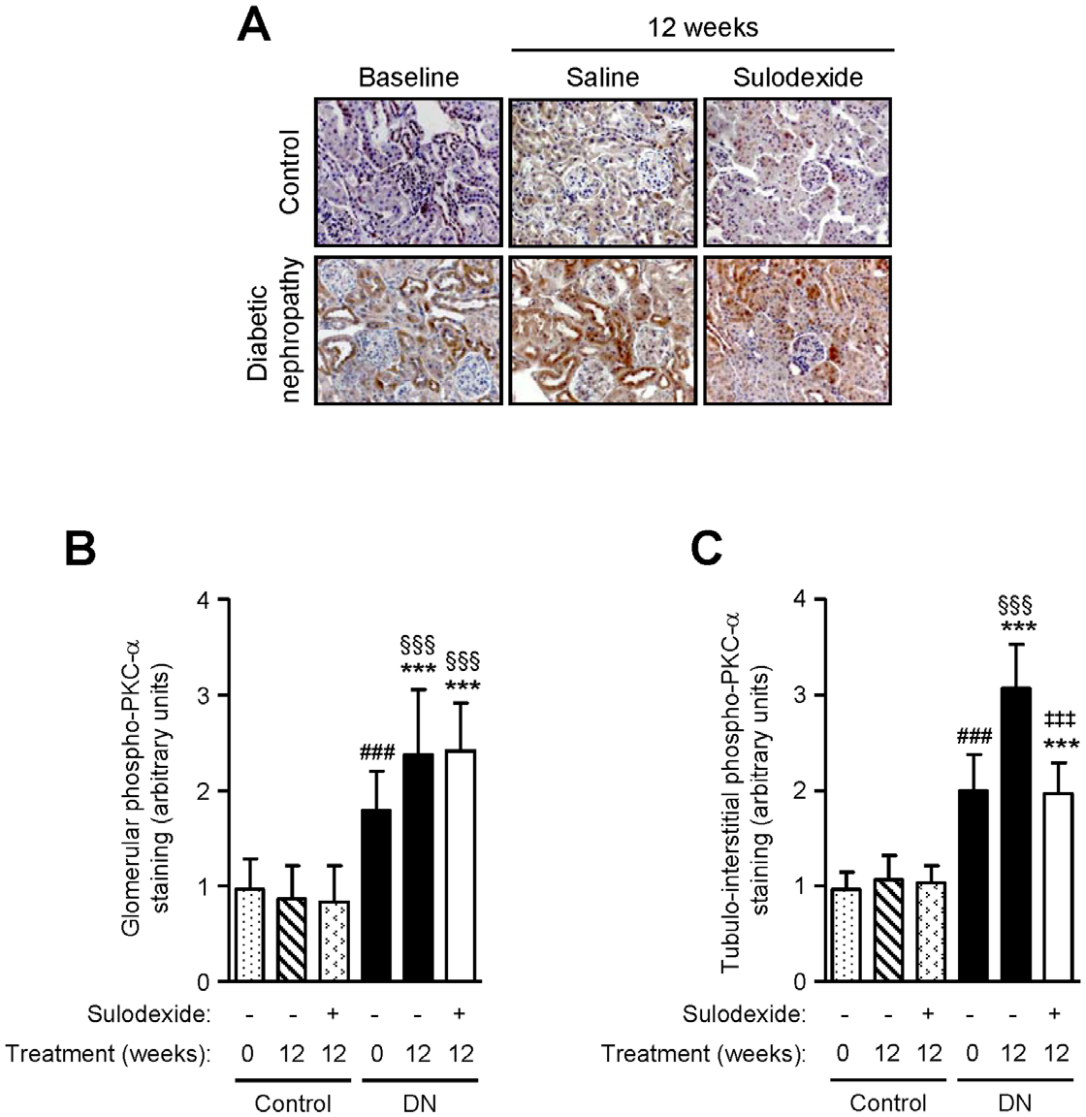
The effect of sulodexide on phosphorylated PKC-α expression in the kidneys of control and DN C57BL/6 mice. Representative images of (A) phosphorylated PKC-α in control and DN mice at baseline and after 12 weeks treatment with saline or sulodexide are shown. Original magnification x1000. Image-based computer assisted analysis was performed to semi-quantify the amount of phosphorylated PKC-α in the (B) glomeruli and (C) tubulo-interstitium of control and DN mice. Results are expressed as mean+SD of data obtained from 6 mice per group. ^§§§^ *P*<0.001, compared to baseline for the same group, ^###^ *P*<0.001, DN baseline vs non-diabetic baseline, ***P<0.001, DN mice vs non-diabetic mice for the same treatment, ^‡‡‡^ *P*<0.001, saline vs sulodexide treatment for the same time-point in DN mice.

**Figure 7:**
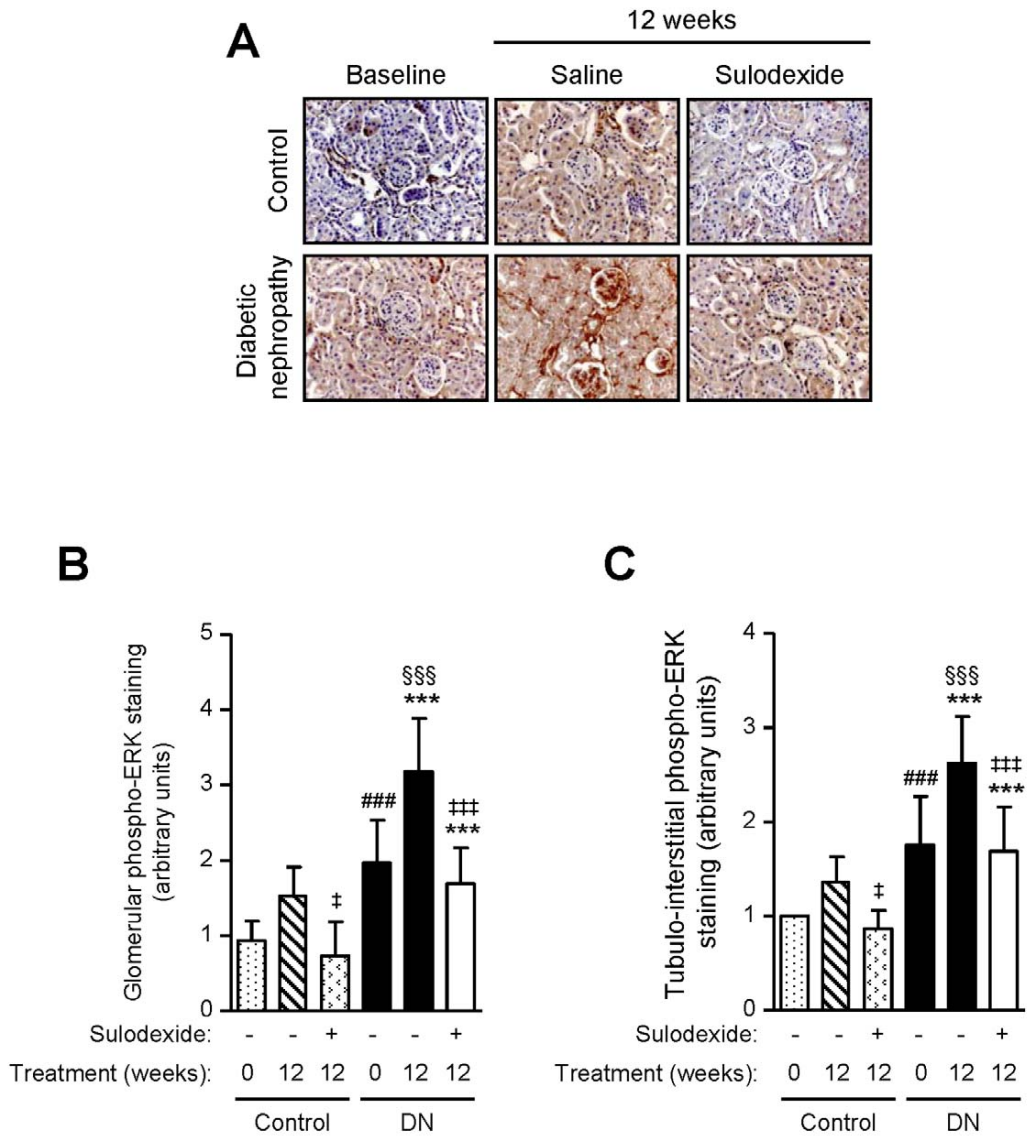
The effect of sulodexide on phosphorylated ERK expression in the kidneys of control and DN C57BL/6 mice. Representative images of (A) phosphorylated ERK in control and DN mice at baseline and after 12 weeks treatment with saline or sulodexide are shown. Original magnification x1000. Image-based computer assisted analysis was performed to semi-quantify the amount of phosphorylated ERK in the (B) glomeruli and (C) tubulo-interstitium of control and DN mice. Results are expressed as mean+SD of data obtained from 6 mice per group. ^§§§^ *P*<0.001, compared to baseline for the same group, ^###^ *P*<0.001, DN baseline vs non-diabetic baseline, ***P<0.001, DN mice vs non-diabetic mice for the same treatment, ^‡^ *P*<0.05, ^‡‡‡^ *P*<0.001, saline vs sulodexide treatment for the same time-point in DN mice.

PKC-α and ERK phosphorylation are signaling pathways involved in matrix protein accumulation in the kidney [27], [28]. Sulodexide treatment did not affect PKC-α phosphorylation but decreased ERK activation in non-diabetic mice (Figures 6 and 7). PKC-α and ERK phosphorylation were increased in the tubulo-interstitium in DN mice when proteinuria became manifest (baseline), and continued to increase over time both in the glomerular and the tubulo-interstitial compartment. Sulodexide treatment reduced ERK phosphorylation in both compartments of the kidney but only reduced tubulo-interstitial PKC-α phosphorylation in DN mice (Figures 6 and 7).

### Effect of Sulodexide on TGF-β1, Fibronectin and Collagen Type I, III and IV Expression

**Figure 8:**
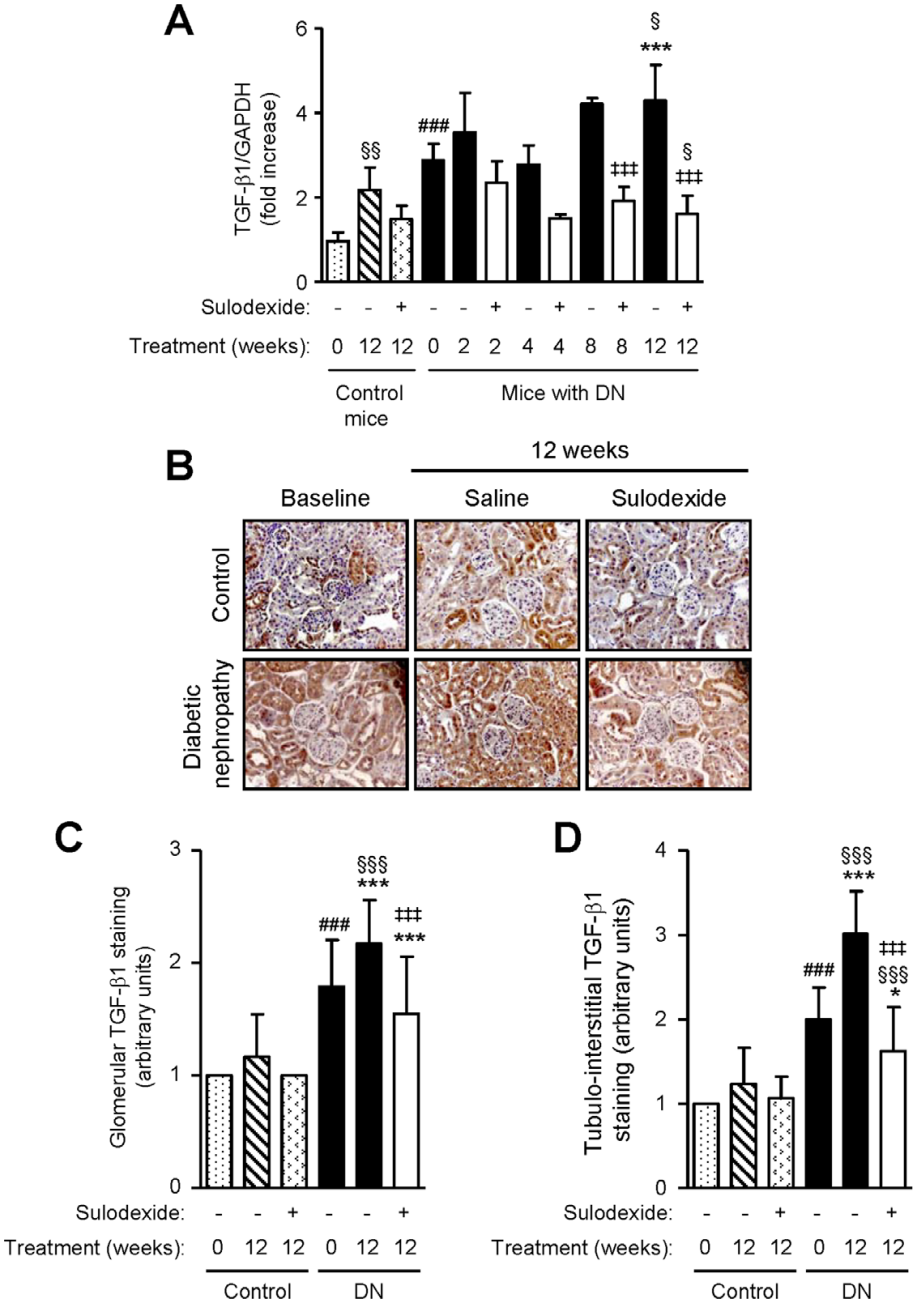
The effect of sulodexide on TGF-β1 gene and protein expression in renal tissue in control and DN C57BL/6 mice. (A) Gene expression of TGF-β1 in control and DN mice treated with saline or sulodexide as determined by real-time PCR. (B) Representative images of TGF-β1 protein expression in control and DN mice at baseline and after 12 weeks treatment with saline or sulodexide are shown. Original magnification x1000. Image-based computer assisted analysis was performed to semi-quantify the amount of TGF-β1 in the (C) glomeruli and (D) tubulo-interstitium of control and DN mice. Results are expressed as mean+SD of data obtained from 6 mice per group. ^§^ *P*<0.05, ^§§^ *P*<0.01, ^§§§^ *P*<0.001, compared to baseline for the same group, ^###^ *P*<0.001, DN baseline vs non-diabetic baseline, *P<0.05, ***P<0.001, DN mice vs non-diabetic mice for the same treatment, ^‡‡‡^ *P*<0.001, saline vs sulodexide treatment for the same time-point in DN mice.

**Figure 9:**
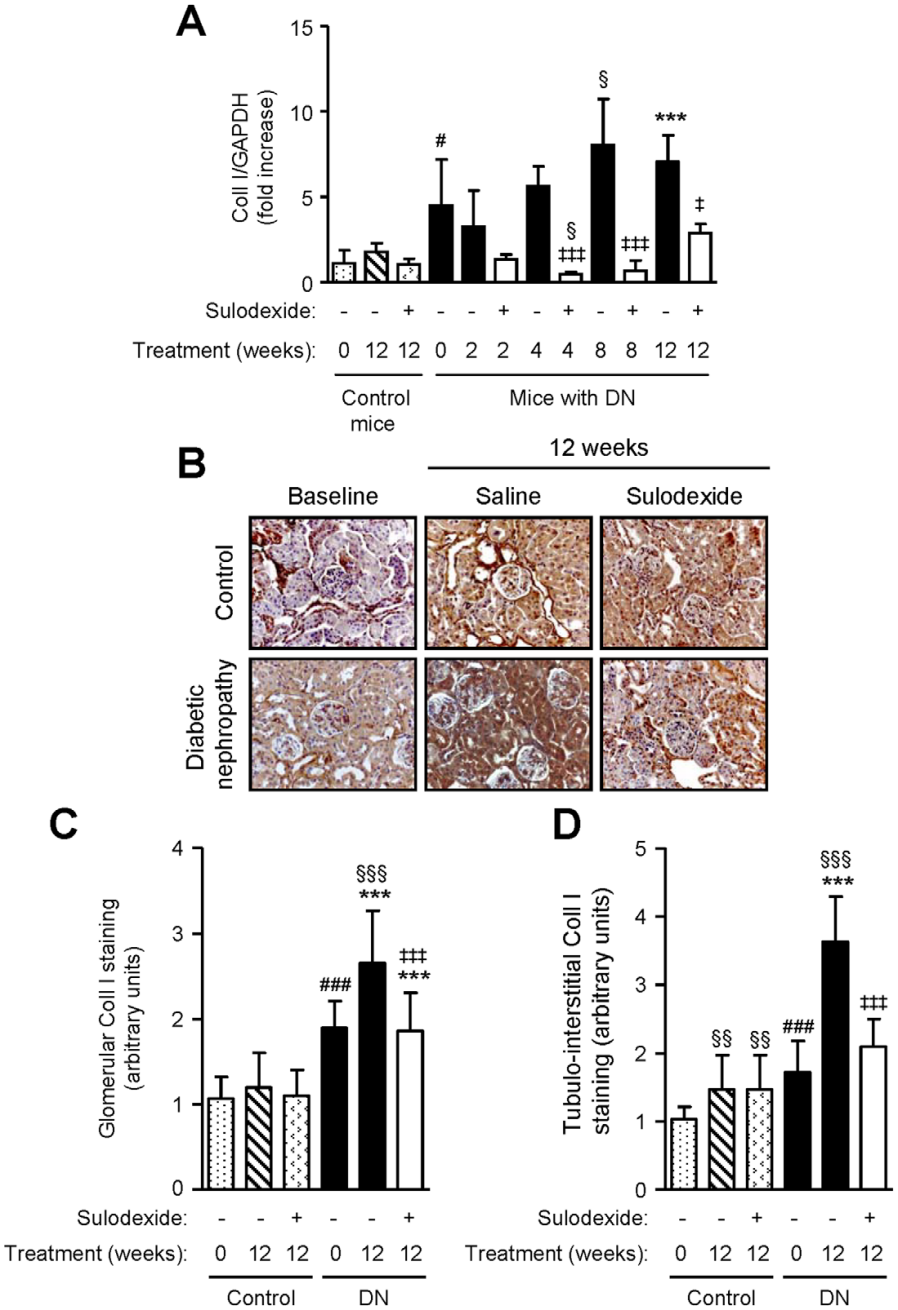
The effect of sulodexide on collagen type I gene and protein expression in renal tissue in control and DN C57BL/6 mice. (A) Gene expression of collagen type I (Coll I) in control and DN mice treated with saline or sulodexide as determined by real-time PCR. (B) Representative images of collagen type I expression in control and DN mice at baseline and after 12 weeks treatment with saline or sulodexide are shown. Original magnification x1000. Image-based computer assisted analysis was performed to semi-quantify the amount of collagen type I in the (C) glomeruli and (D) tubulo-interstitium of control and DN mice. Results are expressed as mean+SD of data obtained from 6 mice per group. ^§^ *P*<0.05, ^§§^ *P*<0.01, ^§§§^ *P*<0.001, compared to baseline for the same group, ^#^ *P*<0.05, ^###^ *P*<0.001, DN baseline vs non-diabetic baseline, ***P<0.001, DN mice vs non-diabetic mice for the same treatment, ^‡^ *P*<0.05, ^‡‡‡^ *P*<0.001, saline vs sulodexide treatment for the same time-point in DN mice.

**Figure 10:**
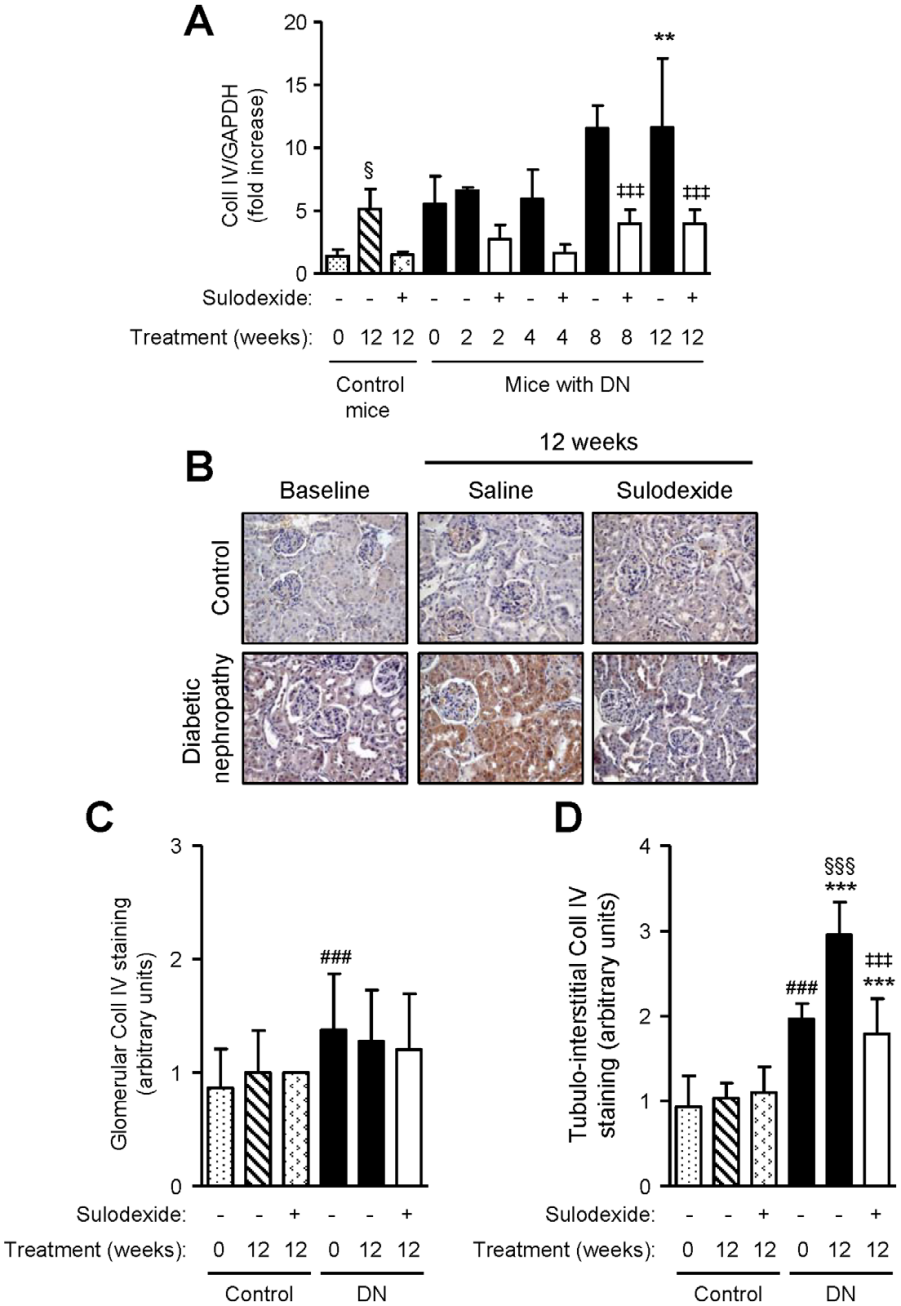
The effect of sulodexide on collagen type IV gene and protein expression in renal tissue in control and DN C57BL/6 mice. (A) Gene expression of collagen type IV (Coll IV) in control and DN mice treated with saline or sulodexide as determined by real-time PCR. (B) Representative images of collagen type IV expression in control and DN mice at baseline and after 12 weeks treatment with saline or sulodexide are shown. Original magnification x1000. Image-based computer assisted analysis was performed to semi-quantify the amount of collagen type IV in the (C) glomeruli and (D) tubulo-interstitium of control and DN mice. Results are expressed as mean+SD of data obtained from 6 mice per group. ^§^ *P*<0.05, ^§§§^ *P*<0.001, compared to baseline for the same group, ^###^ *P*<0.001, DN baseline vs non-diabetic baseline, **P<0.01, ***P<0.001, DN mice vs non-diabetic mice for the same treatment, ^‡‡‡^ *P*<0.001, saline vs sulodexide treatment for the same time-point in DN mice.

DN mice showed higher TGF-β1 mRNA expression than non-diabetic controls, and TGF-β1 protein expression was increased in both the glomerular and tubulo-interstitial compartment of DN mice (Figure 8). Sulodexide treatment did not affect TGF-β1 mRNA or protein expression in non-diabetic mice, but significantly decreased TGF-β1 expression in DN mice (Figure 8A–D). Similar findings were observed with collagen type I and IV mRNA and protein expression (Figures 9 and 10).

**Figure 11:**
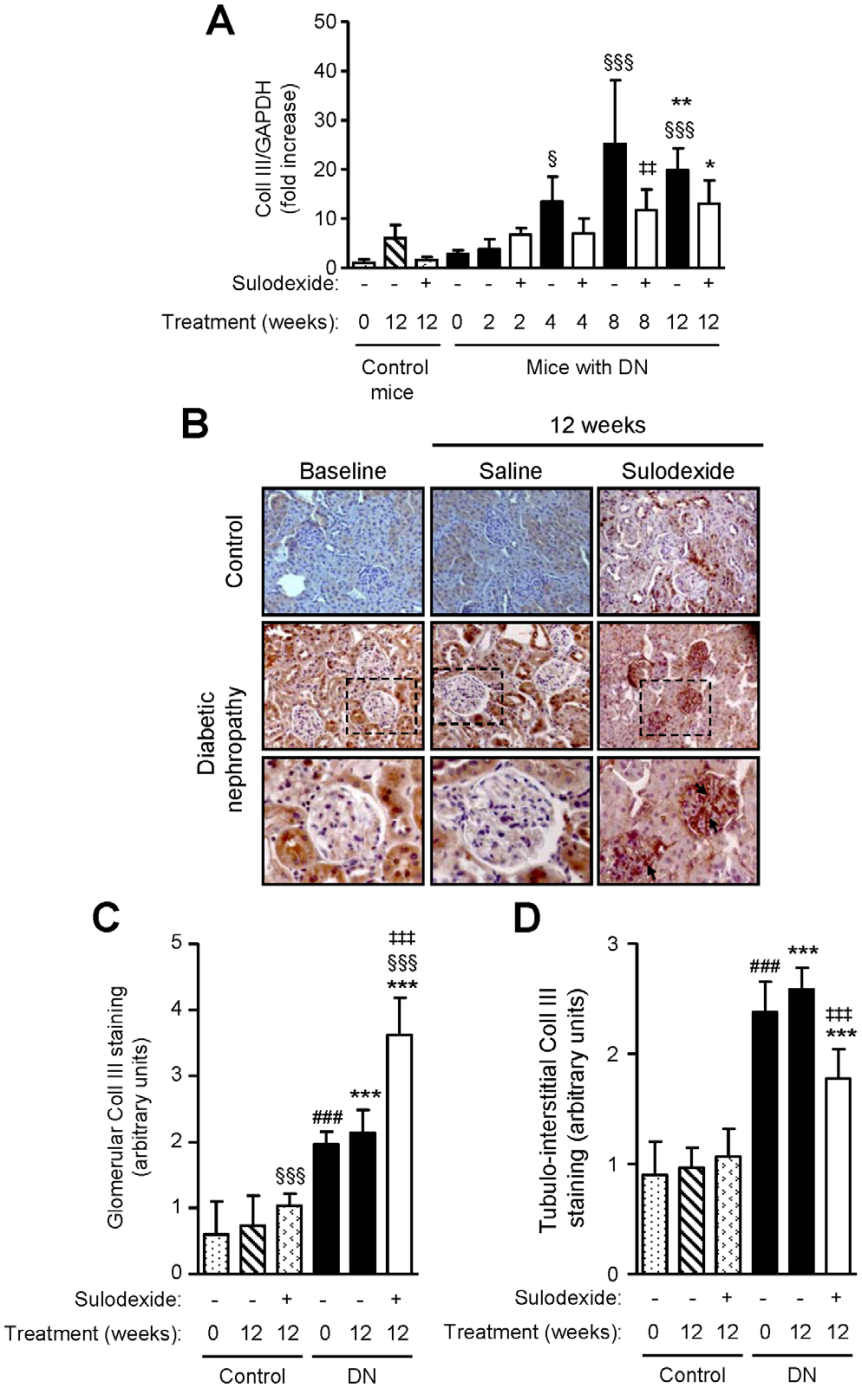
The effect of sulodexide on collagen type III gene and protein expression in renal tissue in control and DN C57BL/6 mice. (A) Gene expression of collagen type III (Coll III) in control and DN mice treated with saline or sulodexide as determined by real-time PCR. (B) Representative images of collagen type III expression in control and DN mice at baseline and after 12 weeks treatment with saline or sulodexide are shown. Original magnification x1000. Boxed areas are enlarged to compare glomerular expression of collagen type III (depicted by arrows). Image-based computer assisted analysis was performed to semi-quantify the amount of collagen type III in the (C) glomeruli and (D) tubulo-interstitium of control and DN mice. Results are expressed as mean+SD of data obtained from 6 mice per group. ^§^ *P*<0.05, ^§§§^ *P*<0.001 compared to baseline for the same group, ^###^ *P*<0.001, DN baseline vs non-diabetic baseline, *P<0.05, **P<0.01, ***P<0.001, DN mice vs non-diabetic mice for the same treatment, ^‡‡^ *P*<0.01,^ ‡‡‡^ *P*<0.001, saline vs sulodexide treatment for the same time-point in DN mice.

**Figure 12:**
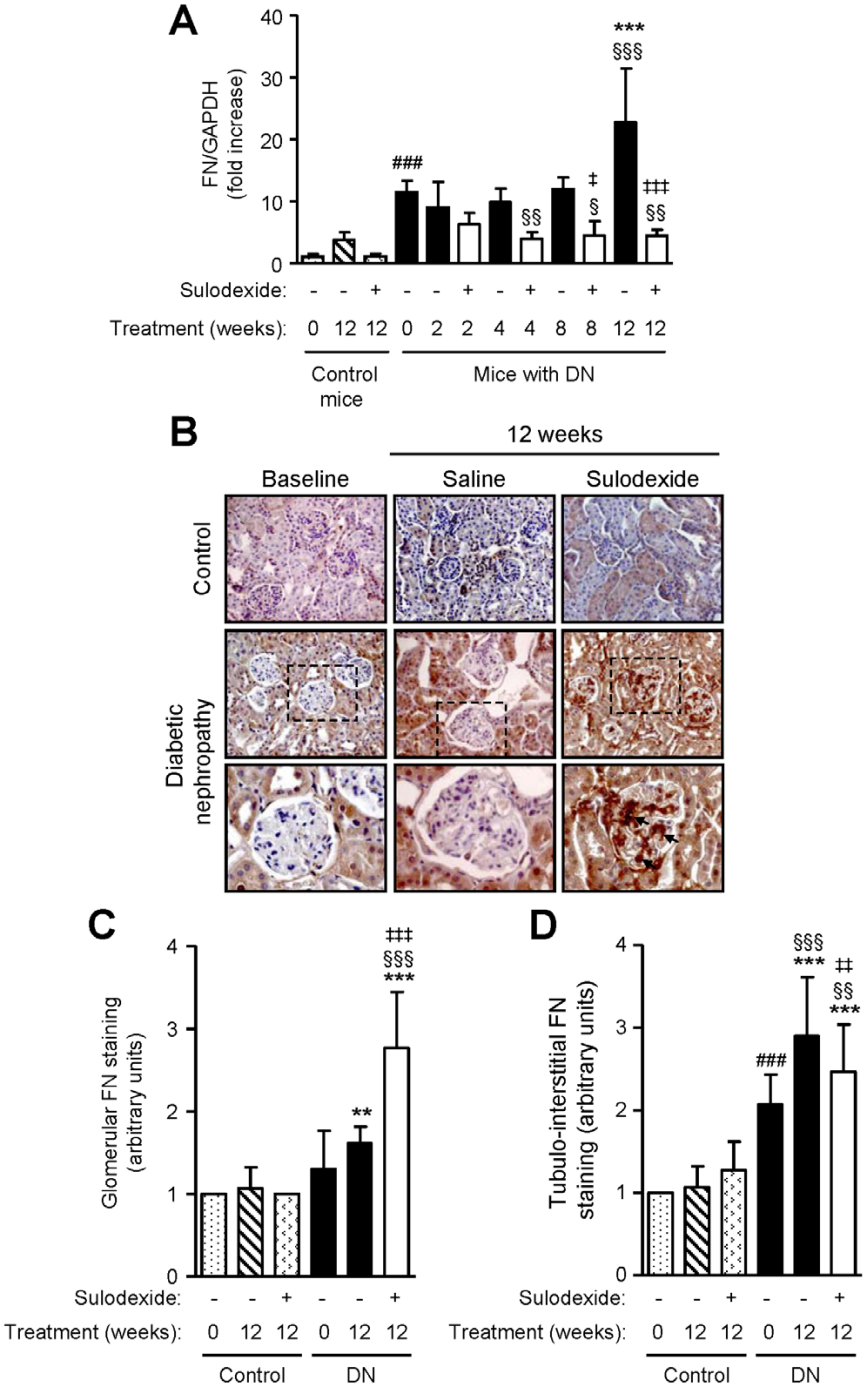
The effect of sulodexide on fibronectin gene and protein expression in renal tissue in control and DN C57BL/6 mice. (A) Gene expression of fibronectin (FN) in control and DN mice treated with saline or sulodexide as determined by real-time PCR. (B) Representative images of fibronectin expression in control and DN mice at baseline and after 12 weeks treatment with saline or sulodexide are shown. Original magnification x1000. Boxed areas are enlarged to compare glomerular expression of fibronectin (depicted by arrows). Image-based computer assisted analysis was performed to semi-quantify the amount of fibronectin in the (C) glomeruli and (D) tubulo-interstitium of control and DN mice. Results are expressed as mean+SD of data obtained from 6 mice per group. ^§^ *P*<0.05, ^§§^ *P*<0.01, ^§§§^ *P*<0.001 compared to baseline for the same group, ^###^ *P*<0.001, DN baseline vs non-diabetic baseline, **P<0.01, ***P<0.001, DN mice vs non-diabetic mice for the same treatment, ^‡^ *P*<0.05, ^‡‡^ *P*<0.01, ^‡‡‡^ *P*<0.001, saline vs sulodexide treatment for the same time-point in DN mice.

DN mice showed increased collagen type III mRNA and protein expression over time, with predominant expression observed within the tubulo-interstitium (Figure 11A–D). Sulodexide treatment reduced mRNA and tubulo-interstitial expression of collagen type III, but markedly increased its expression in the glomeruli (Figure 11A–D). Similar findings were also observed with fibronectin mRNA and protein expression (Figure 12). Collagen type III and fibronectin were weakly expressed in the kidney of non-diabetic mice, their levels remained relatively stable over time and were moderately increased following sulodexide treatment.

### Effect of Gö6976, PD98059 and Sulodexide on Fibronectin and Collagen Type III Synthesis and ERK, PKC-α, PKC-βI and PKC-βII Phosphorylation in MMC

**Figure 13:**
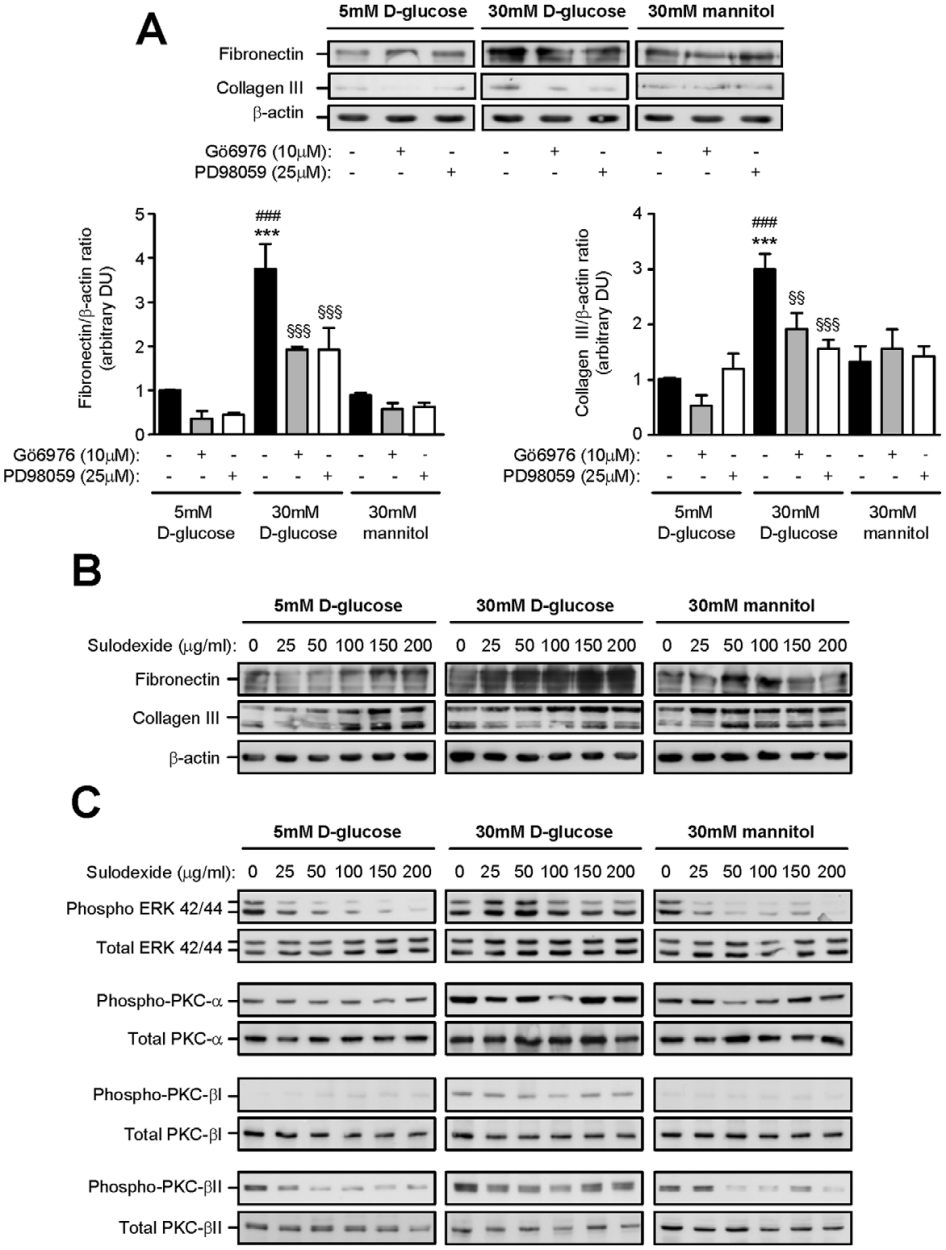
The effect of Gö6976, PD98059 and sulodexide on fibronectin and collagen type III synthesis and phosphorylation of signaling pathways in murine mesangial cells. (A) Western blot analysis showing the effect of 5 mM D-glucose, 30 mM D-glucose and 30 mM mannitol in the presence or absence Gö6976 or PD98059 on fibronectin and collagen type III synthesis in murine mesangial cells after 24 h incubation (upper panel). The intensity of the bands were analyzed by densitometric scan using ImageJ (NIH), normalized to β-actin and expressed as arbitrary densitometric units (DU) (lower panels). ***P<0.001, 5 mM D-glucose vs 30 mM D-glucose, ^###^ *P*<0.001, 30 mM D-glucose vs 30 mM mannitol, ^§§^ *P*<0.01 or ^§§§^ *P*<0.001, with vs without inhibitor for the same stimulation. (B) Western blot analysis showing the effect of 5 mM D-glucose, 30 mM D-glucose and 30 mM mannitol in the presence or absence of sulodexide on fibronectin and collagen type III synthesis in murine mesangial cells after 24 h incubation. (C) Western blot analysis showing the effect of 5 mM D-glucose, 30 mM D-glucose and 30 mM mannitol in the presence or absence of sulodexide on ERK PKC-α, PKC-βI or PKC-βII phosphorylation in murine mesangial cells after 24 h incubation.

MMC constitutively synthesized fibronectin and minor amounts of collagen type III in the presence of 5 mM D-glucose and their levels were not altered when cells were cultured with 30 mM mannitol. Thirty millimolar D-glucose significantly increased fibronectin and collagen type III synthesis compared to 5 mM D-glucose and 30 mM mannitol (Figure 13A). Inhibition of PKC and ERK activation with Gö6976 or PD98059 respectively significantly reduced 30 mM D-glucose induced fibronectin synthesis by 49.53% and 48.81% respectively (*P*<0.001 for both), and collagen type III by 37.12% and 47.96% respectively (*P*<0.01 and *P*<0.001) (Figure 13A).

Under basal conditions, sulodexide increased constitutive expression of fibronectin and collagen type III in a dose dependent manner (fibronectin: 1.52±0.56 vs 1.00±0.00 DU, collagen type III: 2.01±0.75 vs 1.00±0.00 DU, 200 µg/ml sulodexide vs no sulodexide, *P*<0.01 for both), and similar results were also noted when cells were cultured with sulodexide in the presence of 30 mM mannitol (Figure 13B). Concomitant incubation of MMC with 30 mM D-glucose and sulodexide further increased fibronectin and collagen type III synthesis in MMC (fibronectin: 4.03±0.94 vs 1.27±0.62 DU, collagen type III: 2.71±0.82 vs 1.10±0.39 DU, 200 µg/ml sulodexide vs no sulodexide, *P*<0.01 for both) (Figure 11B). ERK, PKC-α, PKC-βI and PKC-βII phosphorylation were increased in cells cultured with 30 mM D-glucose when compared to 5 mM D-glucose or 30 mM mannitol (Figure 13C). Sulodexide decreased ERK and PKC-βII phosphorylation in a dose-dependent manner in control and 30 mM D-glucose stimulated cells but had no effect on PKC-α or PKC-βI phosphorylation.

## Discussion

In this study, C57BL/6 mice developed diabetes mellitus then persistent proteinuria and impaired kidney function after STZ administration. C57BL/6 mice with DN showed predominantly glomerular lesions and proteinuria but relatively mild tubulo-interstitial changes and our results are consistent with previous studies [29], [30]. Glomerular abnormalities included thickening of the GBM, increased accumulation of fibrotic mediators in both the glomerular and tubulo-interstitial compartments, reduced perlecan expression and activation of signaling pathways.

A loss of heparan sulfate proteoglycans in the GBM contributes to proteinuria in glomerular diseases including DN [11], [31] and quantitative and qualitative changes in the *de novo* synthesis of heparan sulfate proteoglycan core protein and/or sulfation pattern of the heparan sulfate glycosaminoglycan chains have been proposed as pathogenic mechanisms [27], [32]. In this study, we demonstrated that perlecan core protein, to which the glycosaminoglycan chains attach, was significantly reduced in the GBM of DN mice despite perlecan mRNA stability. This would suggest that changes to perlecan synthesis in renal cells exposed to elevated glucose concentrations resulted from post-translational modification, a finding that is consistent with previous studies [33], [34]. Although we did not investigate changes to the heparan sulfate glycosaminoglycan chains, given that synthesis of the protein core precedes glycosaminoglycan chain synthesis, a reduction in perlecan core protein expression would also suggest decreased synthesis of heparan sulfate glycosaminoglycan chains and a subsequent reduction in the perm-selectivity of the GBM. The mechanism through which a loss of perlecan expression in the GBM occurs in the setting of DN is currently unknown, but it is possible that elevated glucose mediated induction of TGF-β1 bio-activation may play an important role as observed in mesothelial cells [35]. There is emerging evidence to suggest that reduced intra-glomerular perlecan expression and subsequent albuminuria in DN is also attributed to increased expression of heparanase, an endo-β-D-glucuronidase that plays an important role in the cleavage and degradation of heparan sulfate glycosaminoglycan chains [36], [37]. Glucose-mediated induction of PKC-α phosphorylation has also been shown to play a causative role in the progression of albuminuria since PKC-α deficient diabetic mice showed diminished loss of perlecan expression and were protected from the development of albuminuria [27]. In line with published data, we have also demonstrated that progressive DN in C57BL/6 mice was associated with increased PKC-α activation and heparanase expression that was associated with a concomitant reduction in perlecan expression.

We demonstrated that at the time of established albuminuria, TGF-β1 mRNA level in DN mice was significantly higher than that detected in their age- and sex-matched non-diabetic counterparts. TGF-β1 mRNA level gradually increased with progressive disease manifestations, which paralleled increased intra-renal TGF-β1 protein expression and matrix protein deposition followed by renal fibrosis. Notably at study’s end, mediators of fibrosis were also increased in non-diabetic mice compared to baseline levels, suggesting that advancing age is also associated with increased fibrogenesis.

Preventing the progression of DN and subsequent end-stage renal failure are the fundamental aims in the management of DN. Previous studies have demonstrated that sulodexide or its constituents, namely heparin or dermatan sulfate, can reduce albuminuria in diabetic patients [18], , possibly through its ability to restore heparan sulfate proteoglycans in the GBM, inhibit heparanase and TGF-β1 activity, and reduce collagen type IV deposition in the glomerulus [40]–[43]. Recent multicentre studies however, have failed to reproduce the therapeutic effect of sulodexide [21], [44]. In these large multicentre studies, data from all patients irrespective of race were pooled together and it is possible that any beneficial effect of sulodexide treatment in certain sub-populations may have been lost. Discrepancies between the earlier studies and those of the recent multicentre studies may also be a consequence of differences in treatment duration, recruitment of type I or type II diabetic patients, severity of albuminuria when patients started treatment, rate of absorption of sulodexide from the gastrointestinal tract and drug formulation [44].

There are few mechanistic studies that have investigated the effect of sulodexide on renal histology. We demonstrated a direct and beneficial effect of sulodexide on various disease parameters associated with DN without affecting blood glucose levels. Sulodexide-treated DN mice demonstrated a reduction in albuminuria, serum levels of urea and mesangial expansion that was associated with increased perlecan expression, and down-regulation of ERK phosphorylation, TGF-β1 and heparanase expression, and collagen type I and IV deposition. Our results showed that sulodexide treatment restored perlecan expression to a level similar to that observed in non-diabetic mice. We previously demonstrated that high glucose concentrations induced TGF-β1 which in turn reduced the synthesis of perlecan core protein and heparan sulfate glycosaminoglycan chains in human peritoneal mesothelial cells [35]. These pathogenic mechanisms may also apply in DN, as shown by the inverse relationship between TGF-β1 and perlecan expression in our present study. A reduction in TGF-β1 expression and the replenishment of perlecan may have contributed to the improvement in albuminuria in DN mice following sulodexide treatment. Studies have demonstrated that heparin can inhibit heparanase activity and thus reduce heparan sulfate glycosaminoglycan chain degradation in renal epithelial cells [37]. In this study, sulodexide was shown to reduce heparanase mRNA transcript and protein expression in DN mice to levels detected in non-diabetic mice, and this may have also contributed to the improvement in albuminuria. In addition to its role in the regulation of the perm-selectivity of the GBM, perlecan has also been implicated in angiogenesis, stabilization of the matrix scaffold, and sequestration of growth factors such as FGF [45]. It is therefore possible that the restoration of perlecan in the glomerulus of diabetic kidneys could have various structural and functional benefits.

We demonstrated that sulodexide improved renal histology in DN-treated mice, but further analysis revealed that the effect of sulodexide on signaling pathway activation and matrix protein synthesis was selective. Sulodexide effectively decreased ERK activation and collagen type I and IV mRNA and protein deposition in both glomerular and tubulo-interstitial compartments of the kidney with time, whereas its beneficial effect on PKC-α phosphorylation and collagen type III and fibronectin deposition was only observed within the tubulo-interstitium. Intriguingly, we noted that sulodexide markedly increased glomerular expression of collagen type III and fibronectin in DN mice despite a reduction in gene expression of these two matrix proteins. This may be explained by the fact that cortical tissue was used for our genetic studies, whereby the tissue comprised both glomerular and tubulo-interstitial elements. Given that the tubulo-interstitium occupies up to 90% of the total kidney volume, any changes in collagen type III and fibronectin transcripts in the glomerular compartment following sulodexide treatment may be masked by its effect on the tubulo-interstitium. Since TGF-β1 expression is reduced in DN mice following sulodexide treatment, it is likely that sulodexide-mediated increase in collagen type III and fibronectin expression is through a mechanism that is independent of TGF-β1. Rossini *et al* demonstrated that sulodexide could ameliorate early but not late stages of kidney disease in a murine model of type II DN [46], but in contrast to our studies, these researchers did not report any induction of matrix protein synthesis by sulodexide. This anomaly may be due to different pathogenic mechanisms induced in type I and II DN mouse models and method of sulodexide administration. In a mild non-hypertensive rat model of chronic kidney disease, sulodexide improved renal function, although the beneficial effects of this drug was not sustained [46], an observation that was also observed in our study, whereby serum creatinine levels were reduced after 8 weeks treatment, but subsequently had no effect at later time-points, possibly due to alterations in the structural integrity of the glomerulus following drug treatment.

**Figure 14:**
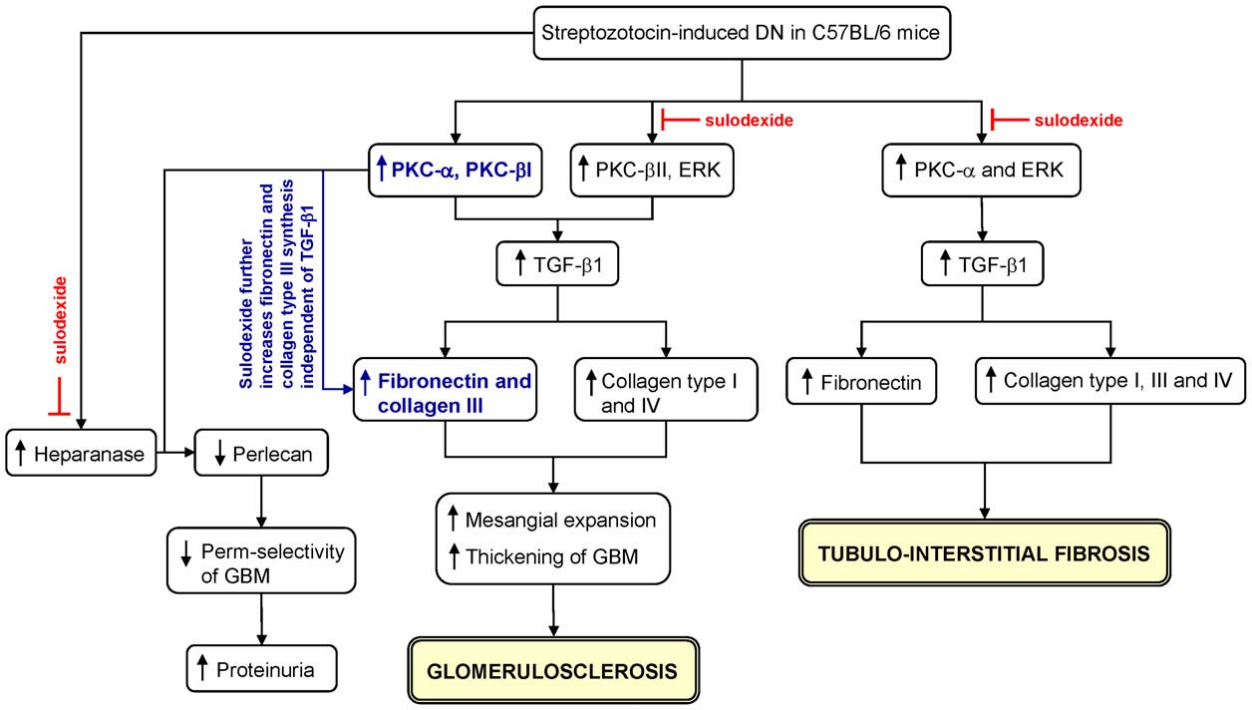
Schematic diagram summarizing the pathways through which glomerulosclerosis and tubulo-interstitial fibrosis are induced in C57BL/6 mice following streptozotocin administration and the differential effect of sulodexide on fibrotic processes.

Although all resident renal cells participate in renal fibrosis, the accumulation of matrix proteins within the glomerulus during pathological conditions is initiated in the mesangium. Mesangial cells were therefore utilized to investigate the effect of sulodexide on matrix protein synthesis *in vitro*. We demonstrated that both PKC and ERK signaling pathways regulated the synthesis of matrix proteins in mesangial cells and reduced phosphorylation of PKC isomers and ERK significantly decreased fibronectin and collagen type III synthesis. Under our experimental setting, MMC constitutively expressed phosphorylated ERK, PKC-α and PKC-βII but not PKC-βI. Elevated glucose concentrations was shown to increase ERK, PKC-α and PKC-βII phosphorylation and induce PKC-βI activation in MMC. The effect of sulodexide on PKC and ERK signaling pathways under physiological and experimental conditions was selective, whereby sulodexide markedly attenuated ERK and PKC-βII phosphorylation in control and 30 mM D-glucose stimulated cells, but had no effect on PKC-α or PKC-βI. These results corroborate our *in vivo* findings. The role of PKC-βI in mediating fibrotic processes in the kidney is well established [47]–[49]. Increased collagen type III and fibronectin synthesis in MMC was observed following their exposure to sulodexide, and their synthesis was further exacerbated by sulodexide in the presence of elevated glucose concentration. Based on these findings, it is plausible to suggest that the observed increase in fibronectin and collagen type III expression in the glomeruli of DN mice was directly attributed to the effect of sulodexide on mesangial cells. A schematic diagram summarizing our *in vivo* and *in vitro* data is shown in Figure 14.

In conclusion, we have demonstrated that sulodexide treatment reduced albuminuria, improved serum levels of urea, restored perlecan expression and ameliorated selective renal histopathologic changes in male C57BL/6 DN mice that included reduced collagen type I and IV deposition, and ERK and PKC-βII activation. In contrast, sulodexide had no effect on PKC-α or PKC-βI activation, but increased glomerular but not tubulo-interstitial deposition of fibronectin and collagen type III. It is possible that an increase in glomerular expression of these matrix proteins and an inability to suppress PKC-α or PKC-βI activation during progressive disease may explain at least in part, why sulodexide showed no efficacy in recent clinical studies although further studies are warranted to confirm this. Whether sulodexide can provide renoprotection in sub-populations of DN patients with specific histopathology remains to be determined.
